# Prenatal stress is a vulnerability factor for altered morphology and biological activity of microglia cells

**DOI:** 10.3389/fncel.2015.00082

**Published:** 2015-03-12

**Authors:** Joanna Ślusarczyk, Ewa Trojan, Katarzyna Głombik, Bogusława Budziszewska, Marta Kubera, Władysław Lasoń, Katarzyna Popiołek-Barczyk, Joanna Mika, Krzysztof Wędzony, Agnieszka Basta-Kaim

**Affiliations:** ^1^Department of Experimental Neuroendocrinology, Institute of Pharmacology, Polish Academy of SciencesKraków, Poland; ^2^Department of Pain Pharmacology, Institute of Pharmacology, Polish Academy of SciencesKraków, Poland; ^3^Laboratory of Pharmacology and Brain Biostructure, Institute of Pharmacology, Polish Academy of SciencesKraków, Poland

**Keywords:** prenatal stress, microglia, inflammation, depressive-like behavior, cytokines, chemokines, neurotrophic factors

## Abstract

Several lines of evidence suggest that the dysregulation of the immune system is an important factor in the development of depression. Microglia are the resident macrophages of the central nervous system and a key player in innate immunity of the brain. We hypothesized that prenatal stress (an animal model of depression) as a priming factor could affect microglial cells and might lead to depressive-like disturbances in adult male rat offspring. We investigated the behavioral changes (sucrose preference test, Porsolt test), the expression of C1q and CD40 mRNA and the level of microglia (Iba1 positive) in 3-month-old control and prenatally stressed male offspring rats. In addition, we characterized the morphological and biochemical parameters of potentially harmful (NO, iNOS, IL-1β, IL-18, IL-6, TNF-α, CCL2, CXCL12, CCR2, CXCR4) and beneficial (insulin-like growth factor-1 (IGF-1), brain derived neurotrophic factor (BDNF)) phenotypes in cultures of microglia obtained from the cortices of 1–2 days old control and prenatally stressed pups. The adult prenatally stressed rats showed behavioral (anhedonic- and depression-like) disturbances, enhanced expression of microglial activation markers and an increased number of Iba1-immunopositive cells in the hippocampus and frontal cortex. The morphology of glia was altered in cultures from prenatally stressed rats, as demonstrated by immunofluorescence microscopy. Moreover, in these cultures, we observed enhanced expression of CD40 and MHC II and release of pro-inflammatory cytokines, including IL-1β, IL-18, TNF-α and IL-6. Prenatal stress significantly up-regulated levels of the chemokines CCL2, CXCL12 and altered expression of their receptors, CCR2 and CXCR4 while IGF-1 production was suppressed in cultures of microglia from prenatally stressed rats. Our results suggest that prenatal stress may lead to excessive microglia activation and contribute to the behavioral changes observed in depression in adulthood.

## Introduction

A growing number of studies indicate that adverse early life experiences may be an important factor in the pathogenesis of depression, due to effects on neurodevelopment (Teicher et al., 2003; de Kloet et al., 2005; Weber et al., 2008). The molecular mechanisms underlying these changes are an active area of investigation.

It has been postulated that stressful events during critical periods of development influence brain by affecting the nervous, endocrine and immune systems. Recent data has shown that changes in the intrauterine environment during the prenatal period, which is critical for growth and neuronal development, might have lifelong effects (Kohman et al., 2008; Diz-Chaves et al., 2013). Among these risk factors stress has been a focus of attention in recent years. According to evidence from epidemiological studies in humans, prenatal stress may rise to behavioral changes including hyperactivity, anxiety, aggression, attention-deficit disorders and cognitive alterations in adolescence and adulthood (Gutteling et al., 2005; O’Connor et al., 2005; Talge et al., 2007). In addition, maternal stress in humans leads to neuro-immuno-endocrine disturbances that enhance susceptibility to some immune-related diseases (e.g., asthma, allergy or diabetes) in adult life (Barker, 2004; Knackstedt et al., 2005).

In rats, the prenatal stress procedure is one of the well-characterized animal models of depression. In this model, increased immobility time in the forced swim test (FST), disturbances in sleep and cognitive functions, decreased sexual behavior and enhanced anxiety-like behavior have been observed (Louvart et al., 2005; Darnaudéry et al., 2006; Maccari and Morley-Fletcher, 2007; Meyer et al., 2008; Szymańska et al., 2009; Budziszewska et al., 2010; Mairesse et al., 2013). Stress during pregnancy in rats also causes long-lasting neurobiological dysfunction, including impaired feedback mechanisms in the hypothalamic-pituitary-adrenal (HPA) axis and the distortion of circadian rhythms (Maccari et al., 2003; Szymańska et al., 2009; Budziszewska et al., 2010). Recent studies have indicated that unfavorable events in early life also affect synaptic plasticity and neurogenesis in the central nervous system (Aisa et al., 2009), which may be related to alterations in the levels of neurotrophic factors (Branchi et al., 2004). Consistent with these observations, our previous study demonstrated that prenatal stress influences the function of the insulin-like growth factor-1 (IGF-1) system. We observed a decrease in IGF-1 level and dysfunction of IGF-1 receptors, as well as dysregulation in the network of IGF-1 binding proteins in the brain of adult rats after the prenatal stress procedure (Basta-Kaim et al., 2014a,b). Some data showed that IGF-1 regulates the immune cell function by influencing the ratio of pro-inflammatory cytokines (Downer et al., 2009; Park et al., 2011) and that its insufficient concentration may enhance the inflammatory response. Consistent with this, prenatally stressed animals have an elevated pro-inflammatory status, which is characterized by the up-regulation of IL-1β, TNF-α and IFN-γ and an exacerbated response to an inflammatory challenge in the hippocampus and frontal cortex of male adult rats (Branchi et al., 2004; Szczesny et al., 2014). Interestingly, changes in some cytokine levels were detected in brain areas of young offspring, suggesting pro-inflammatory orientation in the young immune system (Vanbesien-Mailliot et al., 2007). In fact, in our preliminary study we observed that in young (7-day-old) prenatally stressed offspring the microglia activation was enhanced as evidenced by an increased CD40, MHCII and Iba1 expression. Moreover, the proinflammatory cytokines (especially IL-1β, IL-18) expression was potentiated (our unpublished data).

Numerous studies suggest that maternal stress impairs communication between the immune, endocrine and central nervous systems in developing offspring, which may promote inflammatory processes in adulthood (Markham and Koenig, 2011). The link between inflammatory processes and disturbances in neuron-glia interactions, particularly the activation of microglia, is an active area of research.

Microglia are myeloid cells that are the primary component of the brain’s immune system. They are a good candidate for inducing long-term changes within the brain because these cells have the capacity to become and remain sensitized (Town et al., 2005; Branchi et al., 2014). In response to stress or immune stimulation, microglia up-regulate a number of surface proteins, (CD40, MHC II), cytokines (IL-18, IL-1β, TNF-α, IL-6) and neurotoxic mediators, such as nitric oxide (NO), prostaglandin (PG), E2 and superoxide anions (Kierdorf and Prinz, 2013). These factors initiate both repair and cytotoxic processes via interactions with other brain cells, e.g., astrocytes and neurons. It has been suggested that the sensitized state of “primed” microglia may be prolonged following the initial activation by stimuli, such as injury or stress. Because microglia are believed to be long-living cells, glial pathology may significantly alter neuronal function (Bilbo et al., 2012). Evidences from imaging, cellular and electrophysiological approaches indicate that microglia affect synaptic maturation, remodeling, activities and plasticity as well as neuronal activity in the developing and mature brain (Wu et al., 2013). In the brains in various regions, especially the cortex, hippocampus and cerebellum microglia are involved in the regulation of network activity by controlling the integration of newly born neurons into the existing circuits and elimination of supernumerary neurons (Sierra et al., 2010; Paolicelli et al., 2014). The proposed mechanisms comprise the phagocytosis of non-apoptotic neural precursors and newborn neurons and the release of trophic factors promoting neuronal survival (Wakselman et al., 2008; Ueno et al., 2013). Moreover, during pre- and postnatal brain development, microglia actively engulf synaptic structures and have a major role in controlling the number of synapses through the so-called “synaptic pruning” (Kettenmann et al., 2013).

Based on these studies, it can be suggested that prenatal stress influences microglia function and activity, leading to neuroinflammation and behavioral changes in adult animals. We have previously shown that prenatally stressed adult male rats exhibit depressive—and anxiety—like behaviors. Using this model, we examined the mRNA expression of C1q (complement component 1 q), CD40 (cluster of differentiation 40) and the number of microglia expressing Iba1 (ionized calcium binding adaptor molecule 1) in the frontal cortex and hippocampus, two structures that play a crucial role in the pathogenesis of depressive disorders. Furthermore, we investigated the effect of prenatal stress on the morphology of microglial cells in culture. To study the mechanisms underlying the impact of prenatal stress on the biological activity of cells, we evaluated the following: the expression of mRNA for CD40 and MHC II (major histocompatibility complex class II); synthesis and release of NO; the expression of pro-inflammatory cytokines, including TNF-α (tumor necrosis factor α), IL-1β (interleukin 1β), IL-6 (interleukin 6) and IL-18 (interleukin 18); chemokines, including CCL2 (MCP-1; monocyte chemoattractant protein-1), CXCL12 (SDF-1; stromal cell-derived factor 1) and their receptors (CCR2, CXCR4). Finally, we investigated the release of neurotrophic factors, including IGF-1 and BDNF (brain derived neurotrophic factor), in microglial cells in cultures in an animal model of depression.

## Materials and Methods

### Animals

Sprague-Dawley rats (200–250 g upon arrival) were obtained from Charles-River (Germany) and kept under standard conditions (a room temperature (RT) of 23°C, a 12/12 h light/dark cycle and lights on at 8.00), with food and water available *ad libitum*. Two weeks after arrival, vaginal smears were taken daily from female rats to determine the phase of the estrous cycle. On the day of proestrus, females were placed with males for 12 h. After this, vaginal smears were checked for the presence of sperm. On approximately the 10th day of pregnancy, females were randomly assigned to control and stress groups.

All experiments were designed to minimize the number of animals used and were performed in accordance with the National Institutes of Health Guide for the Care and Use of Laboratory Animals. The experiments were also approved by the Local Ethics Committee, Kraków, Poland.

### Stress Procedure

Prenatal stress was conducted as previously described by Maccari et al. (1995). Briefly, from day 14 of pregnancy until delivery, rats were subjected daily to three stress sessions at 09.00, 12.00 and 17.00 h, during which they were placed in plastic boxes (*d* = 7 cm; *l* = 19 cm) and exposed to a bright light (150 W) for 45 min. Control pregnant females were left undisturbed in their home cages. For *in vitro* experiments, 1–2 day old male offspring were selected from litters. For *in vivo* studies, 3-month old male offspring were chosen. Twenty animals per group (control and experimental) were used for experiments. They were housed in groups of five animals per cage (1–2 animals from each litter) under standard conditions. The offspring of control (unstressed) and stressed mothers were first tested for behavioral changes at 3 months of age.

### Behavioral Studies

#### Sucrose Preference Test

Behavioral change, a reluctance to drink sweetened water (anhedonia), was assessed using a sucrose preference test. For a two-bottle sucrose preference test, prenatally stressed and control male rats were trained to consume a 1% sucrose solution in three 1-h long sessions (from 09.00 until 10.00 am) at 3-day long intervals. In each training session, the positions of the water and sucrose bottles were switched to eliminate any placement preference effect. One week after the last adaptation training session, animals were deprived of food and water overnight and the actual sucrose preference test was performed according to Willner et al. (1987).

Preference was calculated according to the following formula:

% Preference = [(Sucrose preference/Total fluid intake) × 100].

#### Forced Swim Test (FST)

The FST occurred approximately 3 months after prenatal stress. Each animal was individually subjected to two trials during which they were forced to swim in a cylinder (40 cm high, 18 cm in diameter) filled with water (25°C) up to a height of 35 cm. There was a 24-h interval between the first and second trials. The first trial lasted 15 min, while the second trial lasted for 5 min. The observer measured the total durations of immobility, mobility (swimming and climbing) during the second trial (Detke et al., 1995).

### Biochemical Studies

#### Tissue Collection

Forty-eight hours after behavioral testing, animals (3-months of age—adult) were sacrificed under non-stress conditions by rapid decapitation. The hippocampi and the frontal cortices were dissected out on ice-cold glass plates, then frozen on dry ice and stored at −80°C for further biochemical studies.

#### Sample Preparation

Preparation of whole cell extracts for Western blots was conducted according to the method we previously described (Budziszewska et al., 2010). Briefly, tissues in ice-cold RIPA buffer, containing 100 μl of Phosphatase Inhibitor Cocktail 1 and 2, 100 μl of Protease Inhibitor Cocktail, 50 μl of PMSF and OVS up to a total volume of 5 ml (all reagents, Sigma Aldrich, USA), were homogenized using a TissueLyzer II (Qiagen, USA). Samples were shaken in an ice bath for 30 min, centrifuged at 14,000 rpm for 20 min at 4°C and the supernatants were collected. Protein concentrations in the lysates were determined by the method described in Lowry et al. (1951). The cell extract concentrations were standardized by dilution with lysis buffer to the lowest protein concentration obtained. For quantitative RT-PCR, freshly isolated hippocampus and frontal cortex tissue samples were immediately placed in RNALater® solution (Applied Biosystems, USA) and stored at −80°C prior to total RNA extraction.

### Cell Culture

Cultures of microglial cells were prepared from the cortices of 1–2-day old Sprague-Dawley male rat pups with modifications to the method described by Zawadzka and Kaminska (2005). Briefly, after decapitation, brains were immediately removed and cerebral cortices were cut into small pieces (approximately 1 mm). The minced tissue was incubated in HBSS (Hank’s Balanced Salt Solution, Gibco, USA) dissecting medium, containing glucose, BSA, Hepes and 0.025% trypsin (Sigma Aldrich, USA) at 37°C for 20 min. Trypsinization was stopped by the addition of a trypsin inhibitor from Glycine max (soybean) (Sigma Aldrich, USA). A single cell suspension of the tissue was prepared by gentle mixing with a fire-polished Pasteur pipette. Next, cells were plated at a density of 3 × 10^5^ cells/cm^2^ on poly-L-lysine coated culture 75-cm^2^ flasks in culture medium consisting of Dulbecco’s modified Eagle medium DMEM with GlutaMax and high-glucose (4.5 g/L) supplemented with heat-inactivated 10% fetal bovina serum (FBS), 100 U/ml penicillin, and 0.1 mg/ml streptomycin (Gibco, USA). The culture medium was changed after 3 days. On the 9th day of being maintained in *in vitro* conditions of 37°C, 95% O_2_ and 5% CO_2_, the flasks were agitated on horizontal shaker (80 rpm for 1 h and 100 rpm for 15 min). After centrifugation (800 rpm for 10 min), cells were resuspended in culture medium. Cell viability was determined by trypan blue exclusion, and cells were plated at a final density of 1.2 × 10^6^ cells/well onto 6-well plates or 2 × 10^5^ cells/well onto 24-well plates. The purity of microglial cell cultures was assessed using the Iba1 antibody (sc-32725; Santa Cruz Biotechnology Inc., USA); more than 96% of cells were Iba1 positive. Two days after plating, the cells were used for experiments. Cell cultures from control and prenatally stressed animals were obtained according to this method. Cultures from both groups (control and prenatally stressed) were grown simultaneously in the same conditions.

### Immunofluorescence Microscopy in Cultures

Morphological changes of microglial cells were determined by immunofluorescence microscopy. For immunofluorescence imaging, microglia obtained from control and prenatally stressed rats were cultured on sterile cover slips in 6-well plates (1 × 10^6^ cells/well). The cells were rinsed with PBS and fixed with 4% paraformaldehyde (Sigma Aldrich, USA), 20 min at RT and washed twice more with PBS solution. The fixed cells were then permeabilized with 0.1% Triton™ X-100 (Sigma Aldrich, USA) in PBS for 30 min at RT, washed with PBS and blocked with 5% goat serum in PBS. The microglial cells were stained overnight at 4°C with an antibody against Iba1 (microglia/macrophage specific protein, sc-32725, Santa Cruz Biotechnology Inc., USA). After washing with PBS/0.1% Triton X-100, cells were incubated for 2 h at RT with the appropriate fluorescent—conjugated secondary antibody (Alexa Fluor, Jackson Immunoresearch, USA). Images were captured using a fluorescence microscope (Zeiss, Germany). We evaluated any morphological changes caused by prenatal stress by visualizing microglial cells with a 20x or 40x objective on a Zeiss microscope (Zeiss, Germany). To quantitatively characterize microglia morphology we used ImageJ program (USA) and automatically measured perimeter and Feret’s diameter of single microglia cells. Feret’s (maximum) diameter, a measure of cell length, is the greatest distance between any two points along the cell perimeter. For the quantification we took 6 random microscopic fields from each group: control and prenatally stressed (Caldeira et al., 2014).

### NO Release Assay in Cultures

NO secreted into microglia culture medium was measured by a Griess reaction performed according to Hwang et al. (2008). Supernatants (50 μl) were collected and mixed with an equal volume of Griess reagent (0.1% N-1-naphthylethylenediamine dihydrochloride and 1% sulphanilamide in 5% phosphoric acid) in a 96-well plate and incubated for 10 min at RT. Absorbance was measured at 540 nm in a microplate reader (Multiscan, Thermo Labsystem, Finland). The data were normalized to the absorbance of control cells (100%) and expressed as a percent of the control ± SD. Data were obtained from five wells per group, per 1 experiment from 3 independent experiments (3 independent cell cultures each established from male offspring of different females) (Jantas et al., 2011).

### Enzyme-Linked Immunosorbent Assay (ELISA) in Cultures

The supernatant was collected from microglia cells (2 × 10^5^ cells/ well in 24-well plate) and analyzed for the levels of tumor necrosis factor-α (TNF-α, R&D System, USA), interleukin-1β (IL-1β, R&D System, USA), interleukin-18 (IL-18, Invitrogen, USA), interleukin-6 (IL-6, USCN Life Science Inc., China), monocyte chemoattractant protein-1 (CCL2/MCP-1, USCN Life Science Inc., China) and stromal-cell derived factor-1 (CXCL12/SDF-1, USCN Life Science Inc., China) in culture medium. Levels were measured using a commercially available enzyme-linked immunosorbent assay kit (ELISA). Briefly, standards or samples (50 or 100 μl) were dispensed into 96 wells coated with rat TNF-α, IL-1β, IL-6, IL-18, CCL2 or CXCL12 antibody and incubated. After extensive washing, HRP-conjugated streptavidin was pipetted into the wells and incubated. The wells were washed and 3,3′,5,5′-tetramethylbenzidine (TMB) was added. The color develops in proportion to the concentration of the measured protein. Each reaction was stopped after 10 min by the addition of a stop solution. The absorbance was determined using the Infinite 200 PRO Detector (TECAN, Switzerland) system set to the appropriate wavelength (nm).

The detection limits were TNF-α: 5 pg/ml; IL-1β: 5 pg/ml; IL-6: 6.2 pg/ml; IL-18: 4 pg/ml; CCL2: 0.064 ng/ml and CXCL12: 0.125 ng/ml. Positive controls for each assay were provided by the manufacturers.

### Western Blot Analyses in Homogenates of Hippocampus and Frontal Cortex and in Cultures

Western blot analyses were conducted as previously described by Basta-Kaim et al. (2011). Briefly, cells were lysed with a RIPA lysis buffer (Sigma-Aldrich, USA) containing protease inhibitors. The cell lysates or tissue homogenates (equal amounts of protein) were mixed with the buffer (100 mM Tris–HCl, 4% SDS, 20% glycerol, 10% 2-mercaptoethanol, 0.005% bromophenol blue, pH = 6.8) and boiled for 3 min before loading onto a gel. Proteins were separated by SDS-PAGE (4% stacking gel and 10% resolving gel) under constant voltage (90 V in stacking gels and 150 V in resolving gels). The gels were transferred to PVDF membrane (Roche Diagnostic, Germany) by electrophoresis at a 70 V constant current for 1 h and 20 min. The membranes were washed twice with pH = 7.5 tris-buffered saline (TBS) and blocked in 5% non-fat milk for 1 h at RT. Membranes were incubated overnight at 4°C in 1% non-fat milk solution with the appropriate primary antibody: anti-iNOS antibody, anti-IGF-1 antibody, anti-BDNF antibody, anti-Iba1 antibody, anti-CCR2 antibody and anti-CXCR4 antibody (sc-650, sc-1422, sc-546, sc-32725; Santa Cruz Biotechnology Inc., USA; ab-32144, ab-2074; Abcam, USA). The blots were washed twice with TBS containing 0.1% Tween-20 (TBST), washed twice with a 1% blocking solution in TBS and finally incubated with a horseradish peroxidase-linked secondary antibody (Santa Cruz Biotechnology, Inc., USA) for 1 h at RT. These membranes were washed four times with large volumes of TBST, the immunoblots were visualized with a chemiluminescence detection kit (Roche Diagnostic, Germany) and β-actin levels were used for normalization. The semi-quantitative analysis of band intensity was performed using the image analyzer LAS-4000 and Multi Gauge software (FujiFilm, Japan).

#### (Real-Time) RT-PCR Studies in Homogenates of Hippocampus and Frontal Cortex and in Cultures

Total RNA was extracted using the RNA Isolation Kit (Applied Biosystem, USA) following the manufacturer’s instructions. RNA concentrations were determined using a Nanodrop Spectrophotometer (ND/1000 UV/Vis; Thermo Fisher NanoDrop, USA). Identical amounts of RNA (1 μg) were reverse transcribed into cDNA using a commercial RT-PCR kit (Applied Biosystem, USA) according to the manufacturer’s instructions. cDNA was subsequently amplified using TaqMan probes and primers for genes encoding: IL-1β (Rn00580432_m1), IL-6 (Rn01410330_m1), IL-18 (Rn01422083_m1), TNF-α (Rn00562055_m1), iNOS (Rn00561646_m1), CD40 (Rn01423590_m1), MHC II (Rn01424725_m1), IGF-1(Rn00710306_m1), BDNF (Rn01484924_m1), CXCL12 (Rn00573260_m1), CCL2 (Rn00580555_m1), CXCR4 (Rn1483207_m1), CCR2 (Rn01637698_s1), C1q (Rn00570480_m1) and the FastStart Universal Probe Master (Rox) kit (Roche, Switzerland). Amplification was carried out using 10 μl of a mixture containing 1×FastStart Universal Probe Master (Rox) mix, cDNA used as the PCR template, TaqMan forward and reverse primers and 250 nM of a hydrolysis probe labeled at the 5′-end with the fluorescent reporter FAM and labeled at the 3′-end with a quenching dye. Thermal cycling conditions were: 2 min at 50°C and 10 min at 95°C followed by 40 cycles at 95°C for 15 s and at 60°C for 1 min. The threshold value (Ct) for each sample was set in the exponential phase of PCR and the ΔΔCt method was used for data analysis. HPRT (Rn01527840_m1) was used as the reference gene.

#### Statistical Analysis

The outcomes of behavioral studies are presented as the mean ± SD (standard deviation). The results of RT-PCR analyses are presented as average fold change ± SD. The ELISA results are presented as pg/ml or ng/ml ± SD. Western blot results are presented as the percent of control ± SD. Statistical analyses were performed using the Statistica 10.0 Software. All group means were compared by a one-way ANOVA test. *P* values of 0.05 or lower were regarded as statistically significant. ANOVA assumptions (normality of variables’ distribution and homogeneity of variances) were checked by Shapiro-Wilk’s and Levene’s test, respectively.

## Results

### The Effects of Prenatal Stress on Sucrose Preference

**Figure 1:**
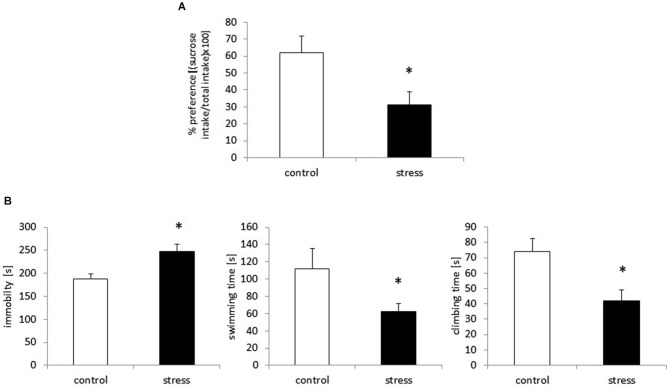
**Prenatal stress leads to depressive-like behavior in adult male rats.** Prenatally stressed rats show anhedonic behavior in sucrose preference test **(A)** the results are expressed as % of preference = [(sucrose intake/total intake) × 100] ± SD. Prenatal stress leads to depressive-like behavior: increased immobility and decreased swimming and climbing time in Forced swimming test **(B)**, the results are expressed in [s] ± SD; *n* = 10 in each group; *p < 0.05; one-way ANOVA.

The sucrose preference test was used to assess anhedonic behavior in the rats. Based on the two-bottle sucrose preference test, prenatal stress caused a significant reduction in sucrose consumption compared to the control group (*F*_1,18_ = 23.42, *p* < 0.05; Figure 1A).

### The Effects of Prenatal Stress on Immobility, Swimming and Climbing Time in the Porsolt Test

Consistent with previous reports (Szymańska et al., 2009; Budziszewska et al., 2010), prenatally stressed rats demonstrated a significantly prolonged immobility time in the FST (*F*_1,18_ = 43.90, *p* < 0.05). Compared with control animals, prenatally stressed rats exhibited shortened swimming time (*F*_1,18_ = 28.70, *p* < 0.05) and climbing time (*F*_1,18_ = 55.67, *p* < 0.05), indicating depressive-like behavior after the prenatal stress procedure (Figure 1B).

### Prenatal Stress Enhances the Expression of Iba1 Protein and C1q, CD40 in Adult Rats

**Figure 2:**
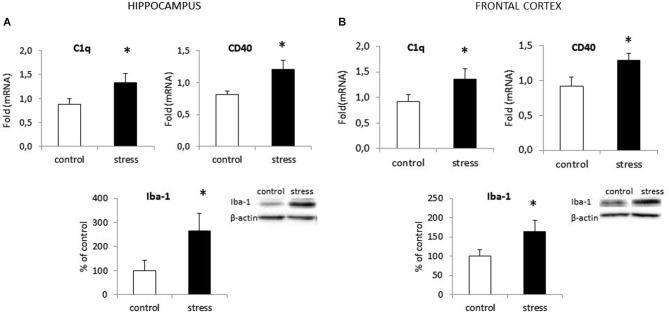
**Prenatal stress enhances the expression of surface markers: CD40 and C1q and protein level of Iba1 in the hippocampus (A) and the frontal cortex (B) in adult rats**. The mRNA expression is presented as the average fold ± SD, *n* = 6 in each group, *p < 0.05; one-way ANOVA. The results from western blot analyses are normalized with β-actin and presented as % of control ± SD, *n* = 6 in each group, *p < 0.05; one-way ANOVA.

In the adult 3-month old offspring of stressed females, we found elevated expression of both C1q and CD40 in the hippocampus (*F*_1,10_ = 24.24; *F*_1,10_ = 42.42) (Figure 2A) and the frontal cortex (*F*_1,10_ = 19.91; *F*_1,10_ = 30.56) (Figure 2B). Moreover, as shown in Figure 2 we found increased Iba1 protein levels in the hippocampus (*F*_1,10_ = 26.93, *p* < 0.05) and the frontal cortex (*F*_1,10_ = 20.32, *p* < 0.05) in prenatally stressed adult rats.

### Prenatal Stress Influences the Morphology of Microglia in Cell Cultures

**Figure 3:**
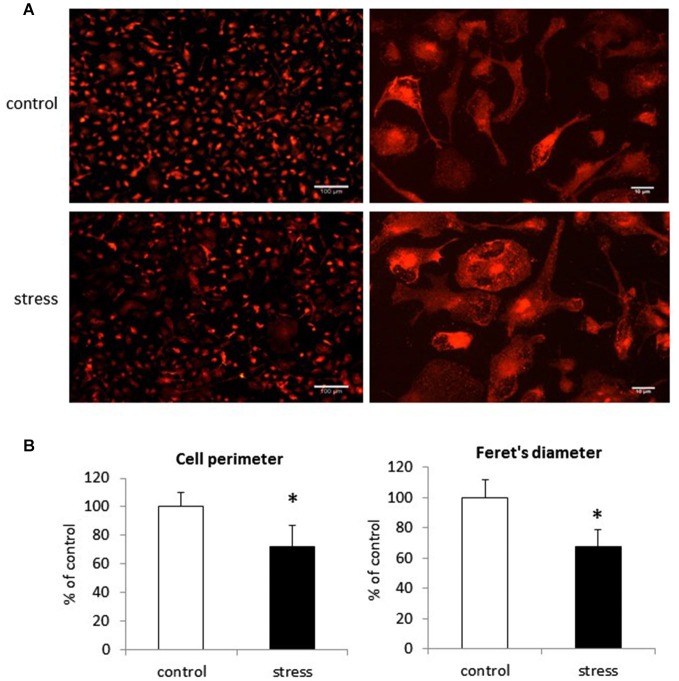
**The impact of prenatal stress on microglial cells morphology in cultures. (A)** Immunofluorescent staining in control cultures and cultures obtained from prenatally stressed rats with Iba1 protein. **(B)** Quantification of microglia morphology changes: perimeter and Feret’s diameter. The results are presented as % of control ± SD, *n* = 6 in each group, *p < 0.05; one-way ANOVA.

In cell cultures obtained from prenatally stressed rats, there were more amoeboid cells than in control cultures. Images from fluorescence microscopy show that prenatal stress influenced microglia branching and cell shape (Figure 3A). Quantitative measurements showed that microglia in cultures from prenatally stressed animals exhibited decreased perimeter as well as the Feret’s maximum diameter. There were more cells with larger soma and shorter cytoplasmic processes in comparison to control cultures where more cells were characterized by ramified morphology (*F*_1,10_ = 23.34, *p* < 0.05; *F*_1,10_ = 26.21, *p* < 0.05). (Figure 3B).

### Prenatal Stress Enhances the Expression of mRNA for Activation Markers in Microglial Cells

**Figure 4:**
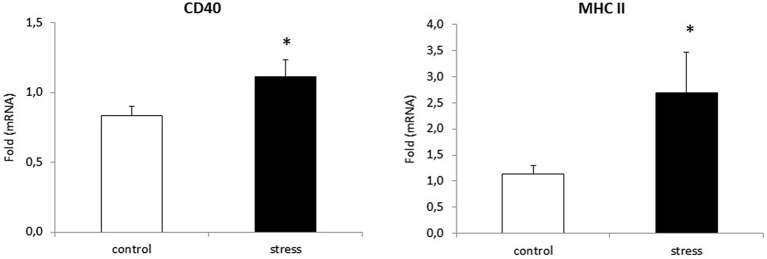
**Prenatal stress enhances the expression of: CD40 and MHC II markers in microglial cell cultures**. The expression of mRNA is presented as average fold ± SD from 3 independent experiments (*n* = 6 in each group), *p < 0.05; one-way ANOVA.

As reported previously, morphological changes in microglia are often accompanied by changes in microglia surface markers, such as CD40 and MHC II (Ling and Wong, 1993). To verify whether prenatal stress affects CD40 and MHC II expression in microglial cells, we performed Real-Time PCR tests. Cells obtained from prenatally stressed rats showed higher levels of mRNA from CD40 (*F*_1,10_ = 26.24, *p* < 0.05) and MHC II (*F*_1,10_ = 22.71, *p* < 0.05) in comparison to cells obtained from control animals (Figure 4).

### Prenatal Stress Modifies NO Production in Microglia Cell Cultures

**Figure 5:**
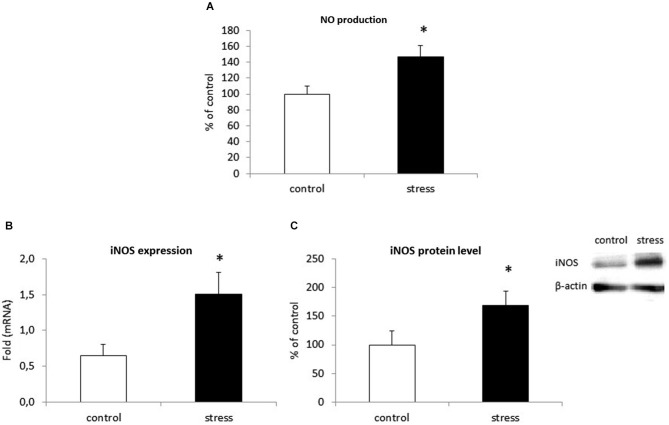
**Prenatal stress increases nitric oxide (NO) production (A), iNOS enzyme expression (B) and protein level (C) in microglial cell cultures.** NO production is expressed as % of control ± SD. The results from qRT-PCR analyses are expressed as average fold ± SD. The results from western blot analyses are normalized with β-actin and presented as % of control ± SD from 3 independent experiments (*n* = 6 in each group); *p < 0.05; one-way ANOVA.

We examined the effect of prenatal stress on NO secretion in microglia by using an assay based on the Griess reaction. As shown in Figure 5A, cells obtained from prenatally stressed rats showed enhanced NO production (*F*_1,28_ = 107.71, *p* < 0.05). To investigate the mechanism by which prenatal stress influences NO production, we determined iNOS mRNA expression and protein levels. Consistent with the up-regulation of NO production, the prenatal stress procedure caused a statistically significantly increase in mRNA expression (*F*_1,10_ = 38.89, *p* < 0.05) and protein (*F*_1,10_ = 22.68, *p* < 0.05) levels of iNOS (Figures 5B,C).

### Prenatal Stress Up-Regulates Pro-Inflammatory Cytokine mRNA Expression and Release by Microglia Cells in Cultures

Prenatal stress significantly increased the expression of pro-inflammatory cytokine mRNA in microglial cells. We observed an increase in IL-1β mRNA (*F*_1,10_ = 65.02, *p* < 0.05), IL-18 mRNA (*F*_1,10_ = 31.06, *p* < 0.05), TNF-α mRNA (*F*_1,10_ = 35.78, *p* < 0.05) and IL-6 mRNA (*F*_1,10_ = 43.7, *p* < 0.05) (Table 1).

**Table 1:** **The effect of prenatal stress on the expression of pro-inflammatory cytokines: IL-1β, IL-18, TNF-α, IL-6, chemokines: CCL2, CXCL12 and chemokine receptors: CCR2, CXCR4 in microglial cell cultures**.

Gene (Fold mRNA)	Control	Stress
IL-1β	0.93 ± 0.13	2.30 ± 0.40*
IL-18	0.80 ± 0.10	1.35 ± 0.22*
TNF-α	0.64 ± 0.15	1.47 ± 0.26*
IL-6	0.74 ± 0.21	1.50 ± 0.18*
CCL2	1.01 ± 0.17	1.38 ± 0.21*
CXCL12	1.03 ± 0.15	1.16 ± 0.12
CCR2	0.93 ± 0.12	1.43 ± 0.29*
CXCR4	1.01 ± 0.15	0.79 ± 0.11*

The results of qRT-PCR analysis expressed as average fold ± SD from 3 independent experiments (n = 6 in each group), *p < 0.05; one-way ANOVA.

**Figure 6:**
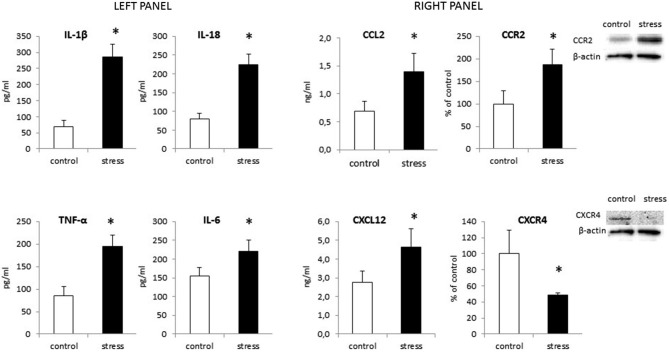
**The impact of prenatal stress on protein levels of cytokines: IL-1β, IL-18, TNF-α and IL-6 (left panel) and chemokines: CCL2, CXCL12 and their receptors: CCR2, CXCR4 (right panel) in microglial cells**. Protein levels obtained from ELISA are shown as pg/ml or ng/ml ± SD from 3 independent experiments (*n* = 8 in each group); The results from western blot analyses are normalized with β-actin and presented as % of control ± SD from 3 independent experiments (*n* = 6 in each group); *p < 0.05; one-way ANOVA.

As shown in Figure 6 (left panel) maternal stress also elevated the release of all cytokines tested in offspring. The most potent increase in cytokine protein levels was in IL-1β (*F*_1,14_ = 193.94, *p* < 0.05) and IL-18 (*F*_1,14_ = 159.75, *p* < 0.05). Prenatal stress also enhanced the production of TNF-α (*F*_1,14_ = 95.33, *p* < 0.05) and IL-6 (*F*_1,14_ = 24.21, *p* < 0.05).

### Prenatal Stress Procedure Modifies Chemokine and Chemokine Receptor Expression in Microglia Cells in Cultures

Our data demonstrate for the first time, that chemokine expression (Table 1) and production (Figure 6 right panel) is altered by maternal stress. In cells cultured from the offspring of females subjected to the stress procedure, we observed increased CCL2 mRNA expression (*F*_1,10_ = 11.25, *p* < 0.05) and release (*F*_1,14_ = 30.55, *p* < 0.05) in comparison to control cultures. We did not detect significant changes in CXCL12 mRNA expression (*F*_1,10_ = 2.51, ns). There was an increase in the secretion of CXCL12 in microglia cultures obtained from pups after the prenatal stress procedure (*F*_1,14_ = 22.35, *p* < 0.05). The CCL2 receptor (CCR2) also showed statistically significant increases in expression (*F*_1,10_ = 15.19, *p* < 0.05) and protein levels (*F*_1,10_ = 21.86, *p* < 0.05) in cells obtained from prenatally stressed rats. In contrast, prenatal stress caused a down-regulation of CXCR4 (CXCL12 receptor) mRNA and protein expression (*F*_1,10_ = 8.12, *p* < 0.05; *F*_1,10_ = 18.58, *p* < 0.05) in primary microglial cells.

### Altered Expression of Neurotrophic Factors in Microglia from Prenatally Stressed Animals

**Figure 7:**
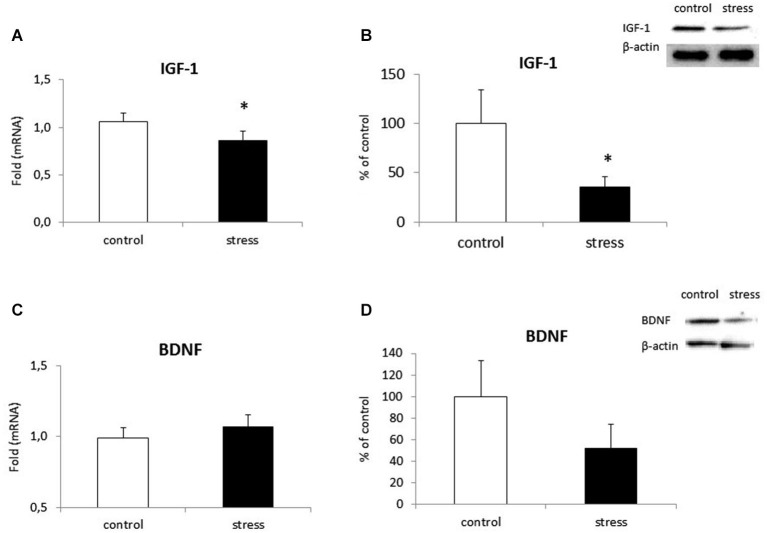
**The impact of prenatal stress on mRNA expression and release of IGF-1 (A,B) and BDNF (C,D) in microglial cells in cultures**. The results of qRT-PCR analyses are expressed as the average fold ± SD. The results from western blot analyses are normalized with β-actin and presented as the % of control ± SD from 3 independent experiments (*n* = 6 in each group), *p < 0.05; one-way ANOVA.

Microglial cells are capable of releasing both inflammatory and neurotrophic factors. Therefore, we examined the effect of maternal stress on the production of IGF-1 and BDNF. Cells obtained from prenatally stressed rats had lower IGF-1 expression (*F*_1,10_ = 13.08, *p* < 0.05) and protein secretion (*F*_1,10_ = 6.51, *p* < 0.05) in comparison to control cells (Figures 7A,B). On the other hand, mRNA expression (*F*_1,10_ = 2.83, ns) and protein release (*F*_1,10_ = 6.34, ns) of BDNF did not differ in microglia exposed to prenatal stress (Figures 7C,D).

## Discussion

Prenatal stress is a well-documented animal model of affective disorders. This procedure has been verified for compliance with the requirements for construct, face and predictive validity by many studies (Maccari and Morley-Fletcher, 2007; Basta-Kaim et al., 2014a,b). This study clearly demonstrates that stress during the critical late period of pregnancy in rats leads to disturbances in the morphology and biological activity of microglial cells.

Data from this study show that NO production, mediated by the enzyme iNOS, and the release of pro-inflammatory cytokines (IL-1β, IL-18, TNF-α, IL-6) are both enhanced in microglia from prenatally stressed animals in comparison to cells from control animals. We also demonstrate, for the first time, that the expression pattern of chemokines (CCL2, CXCL12) and chemokine receptors (CCR2, CXCR4) is altered in microglia obtained from prenatally stressed animals. Microglia in this animal model of depression are impaired in their ability to produce the neurotrophic factor IGF-1. Moreover, in the frontal cortex and hippocampus of adult rats with behavioral disturbances, microglia are activated, as confirmed by the enhanced expression of CD40 protein and increased levels of Iba1.

The data from this study shows that changes in the prenatal environment act as priming factors, which may affect microglial cells. As the primary immune cells in the brain, the aberrant activation of microglia and the impaired secretion of neurotrophic factors may contribute to the onset of affective disorders later in life.

In this study, prenatal stress causes morphological modifications in microglia cultures. These changes result in an increased proportion of amoeboid cells in comparison to control glia, where more cells presented a highly branched morphology. Under physiological conditions microglial cells rapidly change their morphology during early brain development, transitioning from round amoeboid shaped cells to cells with thinner, longer processes and smaller cell bodies. In prenatally stressed animals, this structural remodeling of microglia appears to be altered. Some data suggest that amoeboid cells in young animals reflect immature or over-activated cells and that the dynamic structural remodeling of microglia determines their ability to be re-activated and function in the adult brain (Walker et al., 2014). Recently, it has been shown that in addition to being the first responders to CNS injuries, microglia play an integral role in responding to neuronal activity and regulating synaptic connectivity during development (Cătălin et al., 2013; Harry, 2013; Walker et al., 2014).Therefore, prenatal stress by changing brain microenvironment may affect the function of microglia. Consequently, alterations in the function of these potentially very long-lived cells (Male and Rezaie, 2001) may result in brain disturbances during adulthood.

There is no literature concerning the impact of prenatal stress on microglia morphology in cultures. However, Diz-Chaves et al. (2012) demonstrated that the adult mouse hippocampus contained a small number of short-branched cells and an increased numbers of cells with numerous cell processes (type III). Moreover, Gómez-González and Escobar (2010) used microglial histochemistry to show a reduced number of immature microglia in the two main brain reservoirs of amoeboid cells, the corpus callosum and the internal capsule. The significance of prenatal stress-induced changes in microglia morphology are difficult to explain, but they may reflect a disturbance in remodeling (Gemma and Bachstetter, 2013; Walker et al., 2014).

This study shows that the morphological changes in microglia cultures parallel an enhanced expression of the surface antigens CD40 and MHC II. It is worth emphasizing that C1q and CD40 mRNA expression is enhanced in the frontal cortex and the hippocampus of prenatally stressed adult offspring. On the other hand recent data shown C1q expression also on neurons (Bialas and Stevens, 2013; Stephan et al., 2013), therefore the role of changes in neuronal C1q expression should be also considered. Moreover, in our study western blots show significant increases in Iba1 protein levels in both brain structures. This is consistent with observations that priming glia with the bacteria E. coli led to cognitive impairment in adult rats. Significant memory deficits were only present following subsequent challenges later in life (Bilbo et al., 2008). Moreover, those authors observed a marked increase in the expression of the microglial surface antigens CD40 and MHC II in the hippocampus of adult animals in response to early-life bacterial infection.

It is well accepted that microglial release a variety of soluble mediators that can exert deleterious or beneficial effects on their surroundings. We demonstrate that prenatal stress increases the production of NO and activity of iNOS enzyme in microglia cells. Data on NO action are unequivocal, a majority of them shows a decrease in serotonergic transmission in response to NO release but simultaneously, an inhibitory effect of NO on the monoamine transporters thus contributing to the processes leading to depression (Kiss, 2000). In addition, iNOS inhibitors are effective in depression treatment (Harkin et al., 1999). Some data suggests that the induction and long-lasting activation of oxidative and nitrosative stress pathways may damage the central nervous system (Maes et al., 2013). Moreover, this activation is most frequently accompanied by an inflammatory response. In our study, microglia obtained from prenatally stressed rats displayed a greater ability to release pro-inflammatory cytokines. The expression of IL-1β, IL-18, TNF-α and IL-6 mRNA and protein were increased in comparison to cultures obtained from control offspring. This observation is interesting in the context of our previous studies showing that enhanced pro-inflammatory activity is characterized by the up-regulation of IL-1β, TNF-α and by disturbances in SOCS proteins in the hippocampus and frontal cortex of adult prenatally stressed rats (Szczesny et al., 2014). It has been found that the regulation of inflammation involves changes in gene expression mediated by post-translational modification of histones including acetylation, methylation, phosphorylation, ubiquitination and circullination (Garden, 2013) In fact, it has been found that increased maternal care led to changes in the methylation pattern of IL-10 gene, leading to enhanced IL-10 expression in brain areas and a reduction of morphine evoked addiction behavior (Schwarz et al., 2011). Interestingly, also in depressive patients the altered pattern o DNA methylation including changes in genes IL-6 and C-reactive protein has been demonstrated (Uddin et al., 2010). Taking into account those data it may be postulated that enhanced inflammatory status in prenatally stressed offspring may be at least in part of epigenetic origin. Regardless of the mechanisms responsible for the observed changes, since microglia are the main cells expressing pro-inflammatory cytokines in the brain (Hanisch, 2002; Hinkerohe et al., 2005) and the expression of Iba1 is enhanced in both brain areas of adult rats, microglia are a good candidate for the first cells contributing to an abnormal brain neuronal-immune dialog leading to the development of the behavioral disturbances that are observed in depression.

The results from our behavioral tests are consistent with our earlier reports (Szymańska et al., 2009; Basta-Kaim et al., 2014a,b). In the current study, prenatally stressed rats exhibited an increase in the immobility time along with a decrease in the swimming and climbing behavior measured in the modified Porsolt test. Moreover, the offspring of dams stressed during pregnancy consumed less sweet water than their counterparts from the control group. The sucrose preference test is used as a measure of inability to experience pleasure (anhedonia), one of the core symptoms of depression (Willner et al., 1987; Treadway and Zald, 2011). Because the role of pro-inflammatory cytokines in the induction of depressive-like behavioral disturbances in animal models of this disorder (Maes et al., 2009; Kubera et al., 2011) has already been accepted, therefore we suggest that microglia up-regulation may be one of the main factors responsible for the behavioral deficits observed in adult prenatally stressed rats.

This study demonstrates, for the first time, that prenatal stress up-regulates the release of chemokines (CCL2 and CXCL12) in microglia cultures. Furthermore, prenatal stress modified the expression of the chemokine receptors CCR2 and CXCR4 in cultured microglia cells. CCL2 (MCP-1) is widely expressed in the brain under basal conditions and is up-regulated to recruit monocytes and peripheral macrophages in response to inflammation (Bose and Cho, 2013; Réaux-Le Goazigo et al., 2013; Williams et al., 2014). Therefore, it has been proposed that CCL2 plays an important role in regulating neuroinflammatory activation (Hinojosa et al., 2011; Vogel et al., 2014). Microglia and astrocytes both produce CCL2 in response to pro-inflammatory stimuli (e.g., systemic LPS administration) and the CCL2/CCR2 signaling pathway is apparently crucial for the development of neuroinflammation in the first days following the treatment with this endotoxin (Cazareth et al., 2014; Dinel et al., 2014). Moreover, Thompson et al. (2008) found the down-regulation of pro-inflammatory cytokine production and decreased immune cells activation in the brain of CCL2−/− mice after systemic LPS treatment. Consistent with this study, CCL2 activation of microglia triggers the release of IL-1β and TNF-α (Selenica et al., 2013; Cazareth et al., 2014). The biological effect of CCL2 is mediated largely by activation of the CCR2 receptor, which is found on neuronal and glia cells (Yadav et al., 2010). The concomitant up-regulation of CCL2 release and CCR2 receptor levels we observed suggests that this axis plays a role in neuroinflammation by releasing chemoattractants and activating other immune cells, which exacerbates the inflammatory processes evoked by prenatal stress. Obviously, CCL2 action by other receptors should also be considered.

In addition to the changes in the CCL2/CCR2 system, this study demonstrated that prenatal stress enhances the release of SDF-1 and inhibits the expression of CXCR4 in cultures of microglia.

Data concerning the role of CXCL12 chemokine and their receptors in the brain are scarce and ambiguous. CXCL12 and CXCR4 play a critical role in neurogenesis and neuronal migration patterning during development (Li and Ransohoff, 2008; Triveron and Cremer, 2008; Momcilović et al., 2012). CXCL12 also regulates axonal elongation and branching within hippocampal neurons via interactions with its receptor (Pujol et al., 2005). Given that CXCR4 regulates neuronal system, it may be involved in the establishment of neuron-glia communication and the biological function of microglia. Alternatively, some findings suggest that the stimulation of CXCR4 induces TNF-α release, which may be important in astro-microglial communication (Bezzi et al., 2001). Based on our data from microglia cultures, which have some limitations, we can only conclude that prenatal stress changes the CXCL12/CXCR4 axis while the biological importance of these changes requires further investigation.

Recently, it has been demonstrated that activated microglia are not always toxic. Some contribute to neural repairs and regeneration through phagocytosis and the production of immunoregulatory mediators and growth factors (Suh et al., 2013). Our study demonstrated that priming via prenatal stress decreased the release of IGF-1 but did not affect BDNF levels in microglia. Microglia are the main cell type expressing IGF-1, which has neuroprotective effects along with survival-promoting and pro-regenerative activities (Ness and Wood, 2002; Butovsky et al., 2006; Chhor et al., 2013). We observed in previous studies that adult prenatally stressed rats had lower levels of IGF-1 protein expression in the hippocampus and frontal cortex (Basta-Kaim et al., 2014a,b). Moreover, bidirectional communication between IGF-1 and pro-inflammatory cytokines is impaired in specific brain areas of adult offspring from prenatally stressed dams (Szczesny et al., 2014). It may be postulated that this communication is also disturbed in microglia cultures.

Our study demonstrates, for the first time, that pathological changes in the prenatal environment may act as priming factors and affect the phenotype of microglia cells. These changes may lead to their excessive activation and impaired secretion of pro-inflammatory as well as neurotrophic factors, thus contributing to processes leading to the onset of affective disorders later in life. Further studies are required to determine the precise mechanisms involved in this multi-factorial interaction.

## Conflict of Interest Statement

The authors declare that the research was conducted in the absence of any commercial or financial relationships that could be construed as a potential conflict of interest.
